# T1−/T2‐weighted ratio reveals no alterations to gray matter myelination in temporal lobe epilepsy

**DOI:** 10.1002/acn3.51653

**Published:** 2023-10-23

**Authors:** Colin Denis, Kevin Dabbs, Veena A. Nair, Jedidiah Mathis, Dace N. Almane, Akshayaa Lakshmanan, Andrew Nencka, Rasmus M. Birn, Lisa Conant, Colin Humphries, Elizabeth Felton, Manoj Raghavan, Edgar A. DeYoe, Jeffrey R. Binder, Bruce Hermann, Vivek Prabhakaran, Barbara B. Bendlin, Mary E. Meyerand, Mélanie Boly, Aaron F. Struck

**Affiliations:** ^1^ Department of Neurology University of Wisconsin‐Madison Madison Wisconsin USA; ^2^ Department of Radiology University of Wisconsin‐Madison Madison Wisconsin USA; ^3^ Department of Radiology Medical College of Wisconsin Milwaukee Wisconsin USA; ^4^ Department of Psychiatry University of Wisconsin‐Madison Madison Wisconsin USA; ^5^ Department of Neurology Medical College of Wisconsin Milwaukee Wisconsin USA; ^6^ Department of Biophysics Medical College of Wisconsin Milwaukee Wisconsin USA; ^7^ Department of Medicine University of Wisconsin‐Madison Madison Wisconsin USA; ^8^ Department of Medical Physics University of Wisconsin‐Madison Madison Wisconsin USA; ^9^ Department of Biomedical Engineering University of Wisconsin‐Madison Madison Wisconsin USA; ^10^ William S. Middleton Veterans Administration Hospital Madison Wisconsin USA

## Abstract

Short‐range functional connectivity in the limbic network is increased in patients with temporal lobe epilepsy (TLE), and recent studies have shown that cortical myelin content correlates with fMRI connectivity. We thus hypothesized that myelin may increase progressively in the epileptic network. We compared T1w/T2w gray matter myelin maps between TLE patients and age‐matched controls and assessed relationships between myelin and aging. While both TLE patients and healthy controls exhibited increased T1w/T2w intensity with age, we found no evidence for significant group‐level aberrations in overall myelin content or myelin changes through time in TLE.

## Introduction

The dynamic relationship between brain structure and function is a critical problem to unravel in epilepsy, where structural and functional aberrations can have clinical consequences. Specifically in temporal lobe epilepsy (TLE) patients, the epileptic network has been linked to increases in short‐range functional connectivity.^1^, ^2^ Additionally, recent studies have shown that cortical regions with similar myelin content demonstrate increased functional connectivity,^3^, ^4^ and cortical surface myelin maps from quantitative T1 imaging have revealed increased myelin in the ipsilateral temporal lobe of TLE patients.^5^ This research indicates that myelin is a possible structural correlate of functional connectivity, and thus may be a valuable marker for localizing TLE. This work represents important progress in finding MRI correlates of epileptogenic regions, though there is much to discover about the structural changes caused by epilepsy.

Most investigations to date using noninvasive myelin mapping techniques have focused on cortical myelin.^5^, ^6^ However, subcortical gray matter structures such as the thalamus and basal ganglia are often involved in seizures,^7^ meaning structural changes related to epilepsy are likely not limited to cortex. The ratio of T1‐weighted (T1w) to T2‐weighted (T2w) image intensity, initially proposed by Glasser and Van Essen to reveal cortical surface myelin content,^8^ has also been shown to map myelin in subcortical gray matter.^9^, ^10^ Therefore, we propose to investigate increased T1w/T2w myelin content, both cortical and subcortical, as a marker of pathological structural connectivity in TLE.

Epilepsy may be a progressive disease,^11^ and drug‐resistant TLE patients have been shown to undergo structural and functional brain aging faster than healthy populations.^12^, ^13^, ^14^, ^15^ Myelination is a similarly dynamic process. In their review, Williamson and Lyons discuss how the structure of myelination in human brains changes continually over the course of a lifetime.^16^ We thus hypothesize that TLE patients will display progressive increases in myelination that correlate with age.

In this study, we analyze T1w/T2w structural MR images to investigate potential changes in myelination in TLE patients. We predict that epileptic activity will result in new myelin formation in the seizure focus and in more distant recruited areas. We also investigate how age relates to myelin changes and compare this relationship in TLE patients and healthy controls, hypothesizing increases in myelin content over time in affected regions.

## Method

### Participants

The study sample included 88 participants (51 TLE, 37 controls) from the Epilepsy Connectome Project (ECP). Due to a significant discrepancy in mean age between these groups, we also included 13 controls from the Alzheimer's Disease Connectome Project (ADCP), for a total of 101 subjects. The ECP and ADCP are both collaborations between the Medical College of Wisconsin (MCW) and University of Wisconsin‐Madison (UW). Each study was approved by the respective institutional review boards, and all subjects provided written informed consent. Participant demographics can be found in Table 1.

**Table 1:** Subject demographics.

	TLE patients	Controls
Number	*N* = 51	*N* = 50
Mean age	40.1 ± 11.4 years	39.2 ± 14.2 years
Sex	30 F/21 M (58.8% F/41.2% M)	30 F/20 M (60% F/40% M)
Scan site	19 MCW/32 UW	10 MCW/40 UW
Lateralization	23 left, 13 right, 3 bilateral, 12 unknown	N/A
Mesial temporal sclerosis	9 left, 5 right, 0 bilateral	N/A

Selection criteria included an estimated IQ of at least 70, fluency in English, and age from 18 to 60 years (ECP) or 55 to 90 years (ADCP). Patients participating in the ECP all had a diagnosis of TLE based on clinical, radiographic, and EEG criteria as described in previous papers.^17^, ^18^

### Data acquisition

T1w and T2w structural images were obtained using 3T GE (General Electric) 750 MRI scanners at UW and MCW. T1w scans used a magnetization prepared gradient echo (MPRAGE) sequence (TR = 604 msec, TE = 2.516 msec, TI = 1060.0 msec, flip angle = 8°, FOV = 25.6 cm, 0.8 mm isotropic), and T2w scans used a 3D fast spin‐echo (CUBE) sequence (TR = 2500 msec, TE = 94.398 msec, flip angle = 90°, FOV = 25.6 cm, 0.8 mm isotropic).

### Data preprocessing

T1‐ and T2‐weighted images were preprocessed using standard Human Connectome Project (HCP) pipelines and Statistical Parametric Mapping 12 (SPM).^19^ Volumetric T1w/T2w images were created and processed in SPM using the MRTool toolbox with methods described by Ganzetti et al.^9^, ^10^

We restricted our analyses to cortical and subcortical gray matter using a gray matter mask generated by segmenting each subject's T1w/T2w map into images containing only gray matter. The average of all subjects' gray matter T1w/T2w maps was then subjected to a threshold of 0.2 to form a mask which was applied to all statistical images. This arbitrary threshold resulted in the removal of most white matter and cerebrospinal fluid from T1w/T2w images, but also prevented variations in cortical folding and uncertain gray/white matter boundaries from eliminating voxels which represented gray matter in most, but not all, subjects.

### Statistics

We performed statistical analyses in SPM with a general linear model (GLM) design that included both group membership and age/group interaction as factors. Comparisons of T1w/T2w intensity between patient and control groups used two‐sample (Welch's) unequal variance *t*‐tests. We also used one‐sample *t‐*tests to examine the significance of each group's relationship between T1w/T2w intensity and age.

All reported statistics are corrected for multiple comparisons at the cluster level using the family‐wise error rate (FWER). Reported cluster statistics use a primary cluster‐forming threshold of *p* < 0.001, after which we applied a significance threshold of FWER‐corrected *p* < 0.05. After determining clusters of statistical significance in any test, MNI coordinates of maximally significant results in each cluster were converted into Talairach coordinates using the mni2tal MATLAB function,^20^ and regional labels for these coordinates were subsequently generated with the Talairach Client.^21^, ^22^

## Results

Mean myelin maps in both TLE patient and control groups were broadly in line with findings from Ganzetti et al.,^9^ with the highest T1w/T2w intensity in bilateral basal ganglia. After corrections for multiple comparisons, we found no evidence for any group‐level differences in myelin.

**Figure 1:**
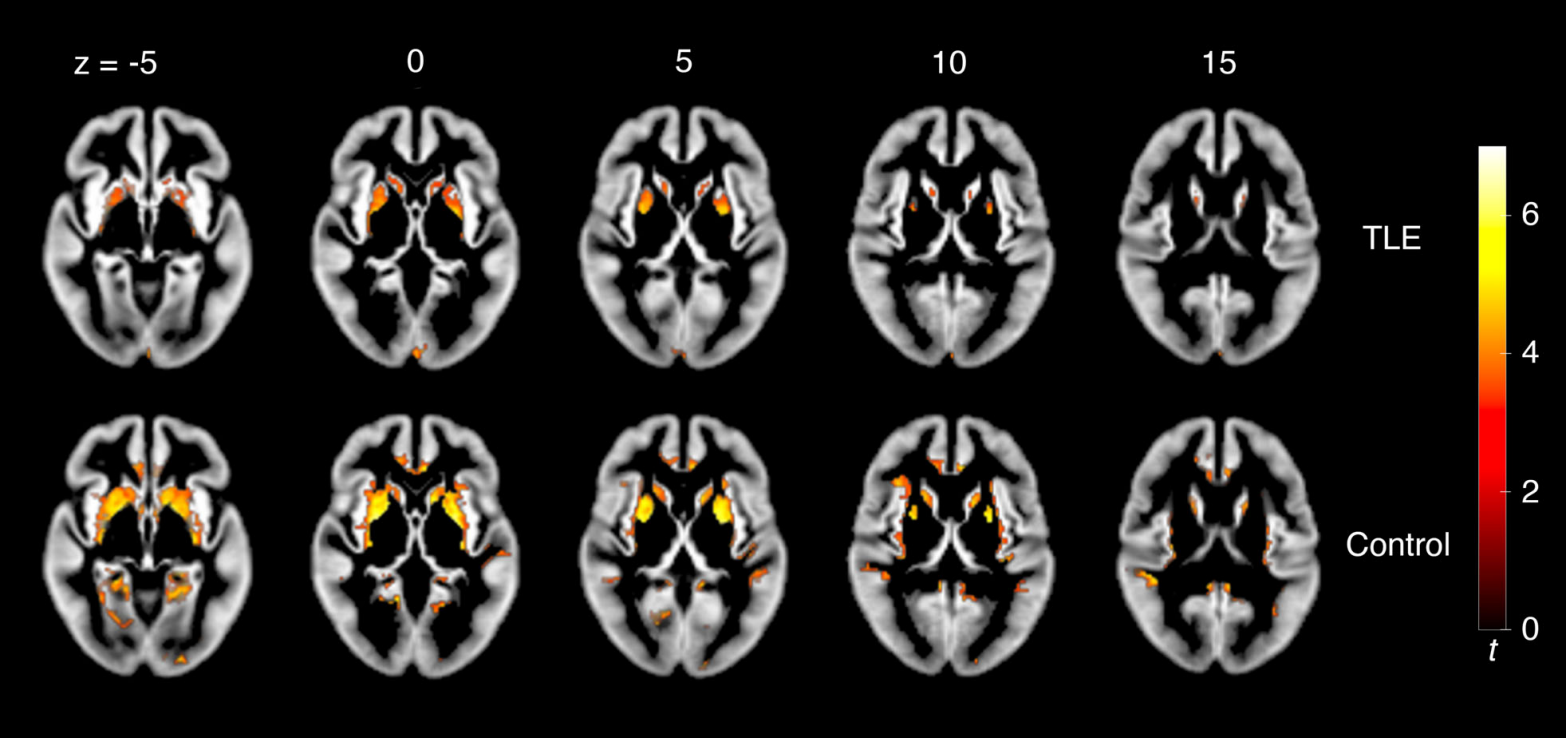
Results of one‐sample *t*‐tests showing a positive linear relationship between T1w/T2w intensity and age in TLE patients and controls. Only results from significant clusters after FWER correction to *p* < 0.05 are displayed at MNI *z*‐coordinates −5, 0, 5, 10, and 15. Findings suggest a lifetime increase in subcortical myelin. While the control group showed a slightly larger overall cluster extent, no significant differences between groups were present.

Both patients and controls show a significant positive relationship between T1w/T2w intensity and age, with effects present primarily in bilateral basal ganglia (Fig. 1). In the TLE group, significant local maxima appeared in the right fusiform gyrus (*p* = 0.002) and the left and right putamen (both *p* < 0.001). In controls, a large significant cluster covered the right orbitofrontal cortex, left putamen, and right parahippocampal gyrus, with each of these local maxima significant at the voxel‐level (*p* ≤ 0.001). However, we found no significant difference in the rate of this age effect between groups. Furthermore, there was no significant relationship between seizure burden and T1w/T2w intensity.

**Figure 2:**
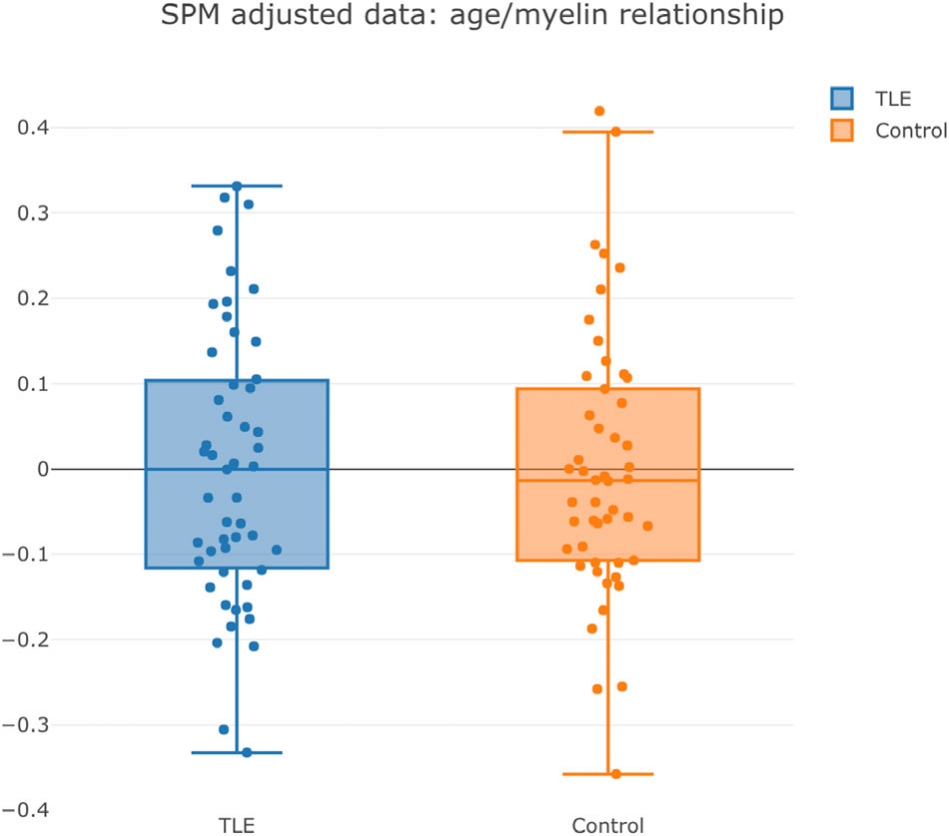
Box plots of SPM‐adjusted voxel data in the parahippocampal gyrus (MNI coordinates 30, −20, −22). Neither group shows a stronger tendency toward outliers, nor is there any significant difference in group variance.

To address concerns that a different cluster extent in the group relationships between age and T1w/T2w may be caused by a greater propensity for outliers in the epilepsy group, we performed a post hoc variance *F*‐test at voxels of maximal significance for the age relationship in patients. These tests revealed no significant group differences in variance, and box plots of the adjusted data at these voxels did not demonstrate any propensity for outliers in either group (Fig. 2).

## Discussion

The controls and patients in this study both show increased T1w/T2w intensity with age, consistent with literature that suggests new myelin formation continues well into adulthood.^16^ However, we find no evidence that TLE patients diverge significantly from myelination patterns exhibited by controls. We anticipated that myelin would increase especially in the epileptic network, which would align with past findings of increased quantitative T1 intensity in the ipsilateral limbic cortex of TLE patients.^5^ Such a pattern could have also represented a structural correlate of connectivity distance reductions found in the epileptic network of TLE patients.^23^

Recent literature supports a hypothesis of increased functional connectivity in the epileptic network.^1^, ^2^, ^23^, ^24^ Sleep slow wave activity related to synaptic potentiation is also increased globally in epilepsy patients, which suggests structural changes due to epilepsy.^25^ These findings inform our hypothesis of increased myelin formation in the epileptic network. Because we interpret the findings of Bernhardt et al. to represent increased myelin,^5^ it is unclear whether our negative results here indicate a lack of difference in myelin content, or a lack of sensitivity of the T1w/T2w myelin imaging method to uncover such differences.

### Limitations

As no noninvasive myelin imaging technique correlates perfectly with myelin content as determined by histology,^26^ it is crucial to understand the sensitivity and specificity of T1w/T2w imaging as a proxy for myelin. Glasser and Van Essen provide a detailed analysis of the value of the T1w/T2w ratio for cortical parcellations, and suggest that the technique shows promise for structural comparisons between healthy and diseased human groups.^8^ Nonetheless, the T1w/T2w ratio has received criticism as a method for quantifying myelin, as it is ultimately a unitless measure which does not quantify any specific physical features.^6^ The method was thus excluded from Mancini et al.'s meta‐analysis of several MRI myelin biomarkers.^26^ Glasser and Van Essen acknowledge that quantitative imaging modalities like T1 mapping may be less sensitive to differences in scan sites and imaging sequences,^6^ though Ganzetti et al. demonstrate that bias correction and intensity scaling can improve reproducibility.^9^ While Uddin et al. find evidence for several non‐myelin structural factors that contribute to the overall T1w/T2w ratio, they also find a significant correlation between T1w/T2w and other MRI proxies for myelin (myelin water fraction, fractional anisotropy, axial diffusivity) across several subcortical regions of interest, and conclude that “myelination contributes to a small but statistically significant portion of T1w/T2w ratio measurements.”^27^

Furthermore, the volumetric image registration methods used in this study may provide suboptimal alignment of cortical tissues.^28^, ^29^ Hinds et al find that surface‐based registration, which accounts for cortical folding, outperforms nonlinear volume‐based registration in cortical alignment quality.^28^ However, volume‐based registration is necessary to align subcortical structures, which are a key component of this study.

Additionally, our hypothesis targets the epileptic network, which manifests in different locations even among patients whose epilepsy is confined to the temporal lobe. While a group‐level analysis between TLE patients and healthy controls allows for greater statistical power to uncover myelin aberrations common to most patients, such an analysis may not offer the sensitivity required to capture aberrations unique to each individual. Thus, highly localized changes to myelination patterns in the epileptic network remain a possibility. Further, in Bernhardt et al.,^5^ all subjects had unifocal epilepsy and went on to surgical resection. The ECP group exhibited a more benign non‐surgical TLE with bilateral and poorly lateralized TLE. It may be that only in relatively severe cases of TLE are myelin abnormalities apparent.

## Conclusion

This work finds no evidence for significant aberrations in myelin formation in TLE patients. These findings do not rule out the possibility of structural correlates to functional connectivity changes in TLE; however, they do suggest that gray matter T1w/T2w intensity values may not efficiently capture any such structural changes if they exist. Future studies using quantitative T1 myelin mapping may be useful to further examine network‐level changes in myelin content.

## Author Contributions

CD, VAN, JM, AN, RMB, LC, CH, EG, MR, EAD, JRB, BH, VP, MEM, MB, AFS contributed to the conception and design of this project. CD, KD, VAN, JM, DNA, AL, AN, LC, CH, MR, EAD, JRB, BH, VP, BBB, MB, AFS contributed to acquisition, analysis, and interpretation of data. CD, LC, JRB, BH, VP, MP, AFS contributed to drafting and revising it critically for important intellectual content. All authors gave their final approval of the version to be published and agree to be accountable for all aspects of the work in ensuring that questions related to the accuracy or integrity of any part of the work are appropriately investigated and resolved.
